# Tailoring inter-core distance of clustered SPIONs using silica spacers for enhanced magnetic particle imaging (MPI)

**DOI:** 10.1039/d6nr00223d

**Published:** 2026-04-09

**Authors:** Elena Ureña Horno, Alexi J. Mores, Lewis D. Owens, Mahon L. Maguire, Harish Poptani, Liam O'Brien, Marco Giardiello

**Affiliations:** a Department of Chemistry, University of Liverpool Liverpool UK magia@liv.ac.uk; b Department of Physics, University of Liverpool Liverpool UK; c Centre for Preclinical Imaging, University of Liverpool Liverpool UK; d Department of Molecular and Clinical Cancer Medicine, University of Liverpool Liverpool UK

## Abstract

An emulsion-based self-assembly strategy for the formation of Superparamagnetic Iron Oxide Nanoparticles (SPION) clusters with precisely tunable silica spacers is reported. Systematic control of the silica shell thickness enables modulation of magnetic inter-core spacing within the clusters. This results in a pronounced enhancement in Magnetic Particle Imaging (MPI) signal generation, attributed to a combination of enhanced long-range magnetic dipole–dipole coupling interaction, the suppression of short-range exchange-like coupling interaction and the passivation of SPION surface spin disorder. The interplay between effects gives rise to an increase in harmonic content of clustered core@shell particles compared with their non-clustered counterparts, resulting in brighter MPI images.

## Abstract 2

Silica spacers tune inter-core interactions in clustered SPIONS, enabling cooperative magnetisation switching and enhanced Magnetic Particle Imaging (MPI) performance.

## Graphical abstract

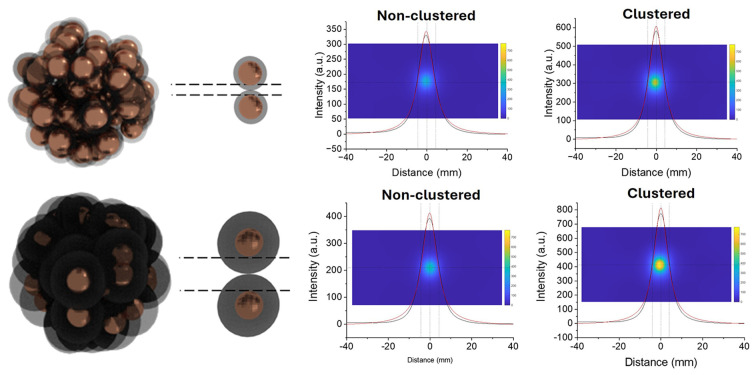

## Introduction

Superparamagnetic iron oxide nanoparticles (SPION) continue to attract considerable attention for biomedical applications such as magnetic resonance imaging (MRI),^1^ drug delivery^2^ and magnetic hyperthermia.^3^ Among these applications, Magnetic Particle Imaging (MPI) has emerged as a promising non-invasive imaging modality that provides quantitative imaging capabilities with zero background signal by detecting the nonlinear magnetization response of SPIONs in an oscillating magnetic field.^4–8^ However, to retain the superparamagnetic state, individual SPIONs are restricted to small core diameters, typically below 20 nm, which limits their overall magnetic moment and, consequently, their detection under field conditions relevant to MPI scanners. This consequently poses a challenge for imaging applications that require high signal strength and strong magnetic response.^9,10^

To enhance the MPI performance of SPIONs while preserving their superparamagnetic behaviour, researchers have developed self-assembly strategies to cluster individual cores into multicore structures, which can improve signal response.^11–17^ However, the magnetic interactions introduced by clustering can play a dual role. While controlled interparticle coupling can enhance nonlinear magnetic response and thus MPI signal generation, uncontrolled aggregation often leads to strong dipole–dipole interactions and, in cases where magnetic cores are in direct contact, short-range exchange-like coupling between neighbouring cores. Such strong interparticle interactions can restrict their independent magnetic moment fluctuations, alter their relaxation dynamics and undermine the superparamagnetic state.^18^ This can reduce the ability of the nanoparticles to respond optimally to the oscillating magnetic fields used in MPI or cause agglomeration. Consequently, clustering can degrade imaging performance and reduce the predictability of the MPI signal response, as observed in our recent studies.^19^

Magnetic interactions between neighbouring SPION cores are sensitive to the centre-to-centre core separation, *d*. In clustered systems, the dominant long-range interaction is magnetic dipole–dipole coupling, for which the interaction energy scales ∝ 1/*d*^3^ in simple models.^20–25^ Consequently, tailoring *d*, relative to the core size, *d*_m_, should lead to pronounced changes in magnetic coupling strength, with direct consequences for magnetic relaxation dynamics and MPI signal generation.

In this work, we present a modular strategy to tailor the nanoscale organisation of SPION clusters by independently controlling silica shell thickness and cluster formation through surfactant-mediated assembly. By introducing silica shells of systematically varied thickness around magnetic cores of identical size, we demonstrate the precise tuning of SPION core-to-core spacing. The silica shells act as chemically inert, non-magnetic spacers that physically separate individual SPION cores within clusters, in principle, reducing the strength of inter-SPION magnetic dipole–dipole interactions.^26–28^ Using this approach, we investigate the impact of interparticle magnetic coupling on MPI signal. While some reports have demonstrated silica shells can reduce the overall magnetisation of composite nanoparticles, due to the increased fraction of non-magnetic material and modification of surface magnetic properties,^29^ we find the silica shells improve MPI response, which we attribute to passivation and a reduction of spin disorder at the SPION surface, as well as mitigating direct short-range coupling, in agreement with prior studies.^30,31^ MPI signal generation is governed by magnetic relaxation dynamics, including Brownian relaxation and tuning of effective magnetic anisotropy.^18,32–34^ This highlights the importance of balancing interparticle interactions and relaxation behaviour when designing MPI tracers. Using an inert spacer, such an approach allows beneficial cooperative magnetic coupling to be tuned, independently of (Brownian) dynamic relaxation, to optimise MPI response. To the best of our knowledge, no previous studies have systematically examined the combined influence of silica shell thickness and clustering on MPI signal generation. Thus, the assembly of silica-coated SPIONs into clusters provides a versatile platform for investigating how spatial organisation and inter-core distance influence magnetic behaviour and MPI performance.

## Results and discussion

### Control of clustering: CTAB optimization at fixed silica thickness

Clusters of silica-coated SPIONs were synthesized *via* a surfactant-mediated assembly method, using cetyltrimethyl ammonium bromide (CTAB) as surfactant. To first isolate the role of CTAB on clustering efficiency, we first constrain silica shell thickness, *t*_S_, while varying CTAB concentration. Individual SPION cores of 15 ± 3 nm were synthesized by thermal decomposition (see SI Fig. S1a).^35,36^ These cores were then coated with silica using a reverse microemulsion method by combining 100 µL ammonium hydroxide and 100 µL TEOS, yielding a uniform silica shell of 20 ± 3 nm thickness.^37,38^ The resulting core@shell nanoparticles measured 55 ± 6 nm in total diameter, as confirmed by TEM (see SI Fig. S1b). These particles are denoted SPION@SiO_2_ (*t*_S_ = 20 nm). X-ray diffraction (XRD) measurements confirmed the presence of both magnetite and silica phases (see SI Fig. S1c). The diffraction peaks correspond well to the standard pattern of Fe_3_O_4_. In the case of SPION@SiO_2_ (*t*_S_ = 20 nm), a broad background signal is observed which masks the magnetite peaks, which is indicative of the presence of amorphous silica.

**Fig. 1:**
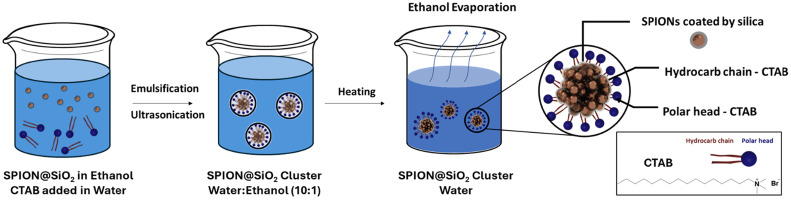
Schematic illustration of the clustering strategy for silica-coated SPIONs. Oleic acid-coated SPION@SiO_2_ (*t*_S_ = 20 nm) nanoparticles dispersed in ethanol are emulsified in an aqueous CTAB solution. Controlled ethanol evaporation under ultrasonic treatment and mild heating promotes surfactant-directed self-assembly within micelles, resulting in stable, well-defined SPION@SiO_2_ (*t*_S_ = 20 nm, *C* = *x*) clusters.

Next, we developed a modified, modular strategy to cluster these silica-coated SPIONs by adapting the assembly method originally reported by Bai *et al.*^39^ Unlike the direct clustering of hydrophobic SPION cores in the original method, our novel approach uniquely clusters SPION@SiO_2_ (*t*_S_ = 20 nm) nanoparticles initially dispersed in ethanol. The subsequent emulsification in aqueous CTAB solutions and controlled ethanol evaporation enables effective surfactant-directed assembly within micelles, resulting in well-defined, stable silica-coated clusters (Fig. 1).

**Fig. 2:**
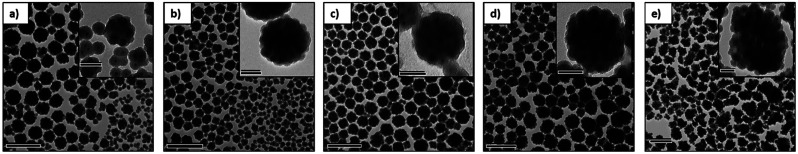
Representative TEM images of SPION clusters SPION@SiO_2_ (*t*_S_ = 20 nm, *C* = *x*), where *x* is the amount of CTAB added in mg. Clusters were assembled at varying CTAB concentrations: (a) SPION@SiO_2_ (*t*_S_ = 20 nm, *C* = 2.5), (b) SPION@SiO_2_ (*t*_S_ = 20 nm, *C* = 5), (c) SPION@SiO_2_ (*t*_S_ = 20 nm, *C* = 10), (d) SPION@SiO_2_ (*t*_S_ = 20 nm, *C* = 20), and (e) SPION@SiO_2_ (*t*_S_ = 20 nm, *C* = 30). Main scale bars are 500 nm and Inset scale bars are 100 nm.

By systematically varying the CTAB concentration during assembly, denoted SPION@SiO_2_ (*t*_S_ = 20 nm, *C* = *x*), where *x* is the amount of CTAB added in mg in the 10 mL solutions, we observed differences in cluster size and uniformity. Structural characterization *via* TEM and DLS confirmed the influence of CTAB concentration. DLS showed an increase in *D*_h_ upon clustering with respect to the non-clustered SPION@SiO_2_ (*t*_S_ = 20 nm) (111.0 ± 0.5). Upon clustering, *D*_h_ was largely uniform across all clustered samples, however with a modest 16% increase in *D*_h_ across the range as higher CTAB concentrations were employed, with all PDIs below 0.15 (Table 1, and SI Fig. S2 for DLS traces). TEM analysis suggested that at low CTAB levels at 2.5 mg (0.686 mM) and 5 mg (1.37 mM), clusters were less uniform and exhibited incomplete particle incorporation, evident by the appearance of individual particles (Fig. 2a and b). Increasing CTAB concentration resulted in fewer individual particles, reaching an apparent optimal concentration at 10 mg (2.74 mM) and 20 mg (5.49 mM) (Fig. 2c and d). At even higher CTAB levels (30 mg, 8.23 mM), clusters appeared once again less organized and less uniform (Fig. 2e), indicating a non-linear relationship between surfactant concentration and clustering. These observations are consistent with surfactant-mediated micelle assembly.^39^ CTAB forms micelles above its critical micelle concentration (CMC; manufacturer reported at ∼1.0 mM in water), which act as a template that facilitate the confinement and assembly of silica-coated SPIONs. At CTAB concentrations near or below the CMC the number of micelles is limited and particle encapsulation is therefore inefficient, thus incomplete clustering is observed. Increasing the CTAB concentration above the CMC increases the micelle population and promotes more efficient cluster formation. However, at higher CTAB concentrations, excess micelles disrupt controlled assembly and lead to less uniform structures observed. This behaviour explains the non-linear relationship between CTAB concentration and cluster uniformity observed in Fig. 2.

**Table 1:** Hydrodynamic diameter (*D*_h_) and polydispersity index (PDI) of SPION clusters measured by DLS as a function of CTAB concentration. All samples were assembled using SPION@SiO_2_ (*t*_S_ = 20 nm) nanoparticles with varying masses of CTAB, denoted (SPION@SiO_2_ (*t*_S_ = 20 nm, *C* = *x*)). Errors reported as ±standard deviation across three scans

Sample	CTAB (mg mM^−1^)	*D*_h_ (nm)	PDI
SPION@SiO_2_ (*t*_S_ = 20 nm)^a^	0	111.0 ± 0.5	0.14
SPION@SiO_2_ (*t*_S_ = 20 nm, *C* = 2.5)	2.5/0.67	156.7 ± 1.3	0.11
SPION@SiO_2_ (*t*_S_ = 20 nm, *C* = 5)	5/1.37	167.7 ± 1.0	0.10
SPION@SiO_2_ (*t*_S_ = 20 nm, *C* = 10)	10/2.74	171.6 ± 0.9	0.10
SPION@SiO_2_ (*t*_S_ = 20 nm, *C* = 20)	20/5.49	181.7 ± 3	0.11
SPION@SiO_2_ (*t*_S_ = 20 nm, *C* = 30)	30/8.23	180.1 ± 3	0.14

a SPION@SiO_2_ (*t*_S_ = 20 nm) measure in ethanol.

To provide initial insight into the MPI properties of the clusters, analysis using the Momentum™ MPI relaxometry module, Relax™, were performed. Relax™ measures sample magnetization (*M*) as a function of the applied field (*H*), without a field gradient to generate an FFP. The module is sensitive to reversal dynamics over both Néel and Brownian timescales, thus is a useful and rapid tool to provide predictive information of tracer performance prior to full MPI studies. Table 2 shows the Relax™ sensitivity and resolution data, which are extracted from the estimated point spread function from Relax™ measurements (see SI Fig. S3). The effect of clustering is quite apparent, with a clear (∼15%) increase in MPI sensitivity and improvement in resolution between individual SPION@SiO_2_ (*t*_S_ = 20 nm) particles and all clustered SPION@SiO_2_ (*t*_S_ = 20 nm, *C* = *x*) samples. Within clustered samples, MPI resolution and signal amplitude remained largely unaffected by CTAB concentration, despite the observed variation in clustering uniformity at differing CTAB concentration. These results indicate that for SPION@SiO_2_ (*t*_S_ = 20 nm), variations in CTAB concentration do not significantly affect MPI sensitivity or resolution. This is perhaps unsurprising given the largely consistent *D*_h_ across all clustered samples and indicates the small variation in core-to-core distance and compaction between clustered samples (for constant shell thickness) does not dramatically alter magnetic response when averaged over the particle population, compared with the broader effect of cluster formation. This finding motivated further investigation into the effect of silica shell thickness on MPI performance, keeping cluster compaction constant.

**Table 2:** Relax™ relaxation data showing resolution (FWHM), and sensitivity (signal amplitude) for non-clustered SPION@SiO_2_ (*t*_S_ = 20 nm) nanoparticles *versus* clustered SPION@SiO_2_ (*t*_S_ = 20 nm, *C* = *x*) particles, where *x* = amount of CTAB added in mg (see SI Fig. S3 for point spread functions)

Sample	Resolution (mm)	Signal amplitude (a.u.)
SPION@SiO_2_ (*t*_S_ = 20 nm)	6.89	6.62
SPION@SiO_2_ (*t*_S_ = 20 nm, *C* = 2.5)	5.94	7.93
SPION@SiO_2_ (*t*_S_ = 20 nm, *C* = 5)	5.99	7.54
SPION@SiO_2_ (*t*_S_ = 20 nm, *C* = 10)	6.01	7.47
SPION@SiO_2_ (*t*_S_ = 20 nm, *C* = 20)	6.06	7.83
SPION@SiO_2_ (*t*_S_ = 20 nm, *C* = 30)	6.00	7.67

### Control of inter-core distance: silica thickness modulation under fixed CTAB conditions

**Fig. 3:**
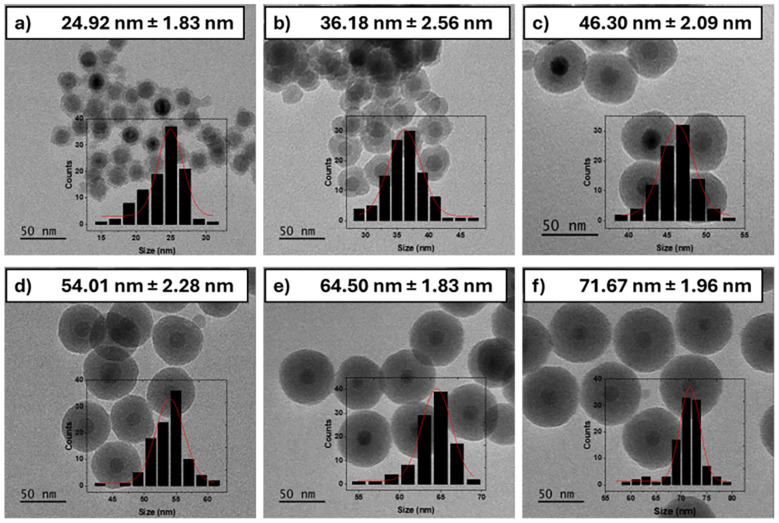
TEM images of SPION@SiO_2_ (*t*_S_ = *y* nm)nanoparticles with increasing silica thicknesses, where y is the thickness in nm, showing *t*_S_ = (a) 4 nm, (b) 10 nm, (c) 15 nm, (d) 18 nm, (e) 23 nm, and (f) 27 nm. Size distribution histograms (inset) were generated measuring over 100 particles for the statistical analysis, with errors reported as ±standard deviation.

Based on the clustering behaviour observed in the CTAB screen, CTAB concentration was fixed at 10 mg (2.74 mM) in subsequent experiments, to maintain consistent *D*_h_ and isolate the effects of silica thickness on inter-core distance. A new batch of SPIONs were synthesized with an average diameter of 17.24 ± 1.43 nm (see SI Fig. S2), and silica shell thickness was modulated by adjusting the relative concentrations of TEOS and ammonium hydroxide in the reverse microemulsion step (see SI Table S1 for synthesis conditions). This approach yielded a series of SPION@SiO_2_ (*t*_S_ = *y* nm) nanoparticles of varying shell thicknesses, *y*, with *t*_S_ = 4 nm; 10 nm; 15 nm; 18 nm; 23 nm; 27 nm, as confirmed by TEM (Fig. 3).

These SPION@SiO_2_ (*t*_S_ = *y* nm) core@silica nanoparticles were then assembled into clusters using the previously optimized CTAB concentration to maintain consistent *D*_h_. Samples are denoted SPION@SiO_2_ (*t*_S_ = *y*, *C* = 10). DLS and TEM measurements (SI Fig. S5 and S6) were performed confirming cluster formation. The measured hydrodynamic diameters, *D*_h_, across the series remained consistent within error, ranging from *D*_h_ ∼ 257–297 nm with no systematic dependence on *t*_S_. All PDIs remained low, (below 0.27) with a trend in reduction of PDI for thicker silica shells particles, suggesting larger particle sizes leads to more monodisperse clusters formation (Table 3).

**Table 3:** Hydrodynamic diameter (*D*_h_) and polydispersity index (PDI) of SPION@SiO_2_ (*t*_S_ = *y* nm, *C* = 10) clusters measured by DLS. All samples were assembled using SPION@SiO_2_ (*t*_S_ = *y* nm) nanoparticles with varying shell thicknesses (*y*), using the same concentration of CTAB (10 mg, 2.74 mM) throughout. Errors reported as ±standard deviation across three scans

Sample	*D*_h_ (nm)	PDI
SPION@SiO_2_ (*t*_S_ = 4 nm, *C* = 10)	297.0 ± 0.4	0.27
SPION@SiO_2_ (*t*_S_ = 10 nm, *C* = 10)	294.1 ± 0.3	0.21
SPION@SiO_2_ (*t*_S_ = 15 nm, *C* = 10)	279.3 ± 0.4	0.22
SPION@SiO_2_ (*t*_S_ = 18 nm, *C* = 10)	284.4 ± 0.5	0.16
SPION@SiO_2_ (*t*_S_ = 23 nm, *C* = 10)	292.5 ± 0.5	0.15
SPION@SiO_2_ (*t*_S_ = 27 nm, *C* = 10)	257.3 ± 0.2	0.15

### MPI analysis

2D MPI images were acquired using the Momentum™ MPI scanner. Data are presented normalised to total sample Fe mass, as determined by ICP-OES, to enable quantitative comparison of signal intensity across all samples. MPI signal and resolution measurements for each silica coated sample, as well as for the non-silica coated sample SPION@SiO_2_ (*t*_S_ = 0 nm), are derived from 2D images and associated line profiles obtained by extracting an *x*-direction profile through the voxel of maximum signal (see SI Fig. S7 for full images and line profiles). The profiles provide the maximum intensity value, *i.e.*, the highest value within the region of interest (ROI), and the total intensity, *i.e.*, total integrated intensity within the ROI area. The line profiles were also fitted with a Lorentzian function to provide the Full Width Half Maximum (FWHM) as a metric for spatial resolution.

**Fig. 4:**
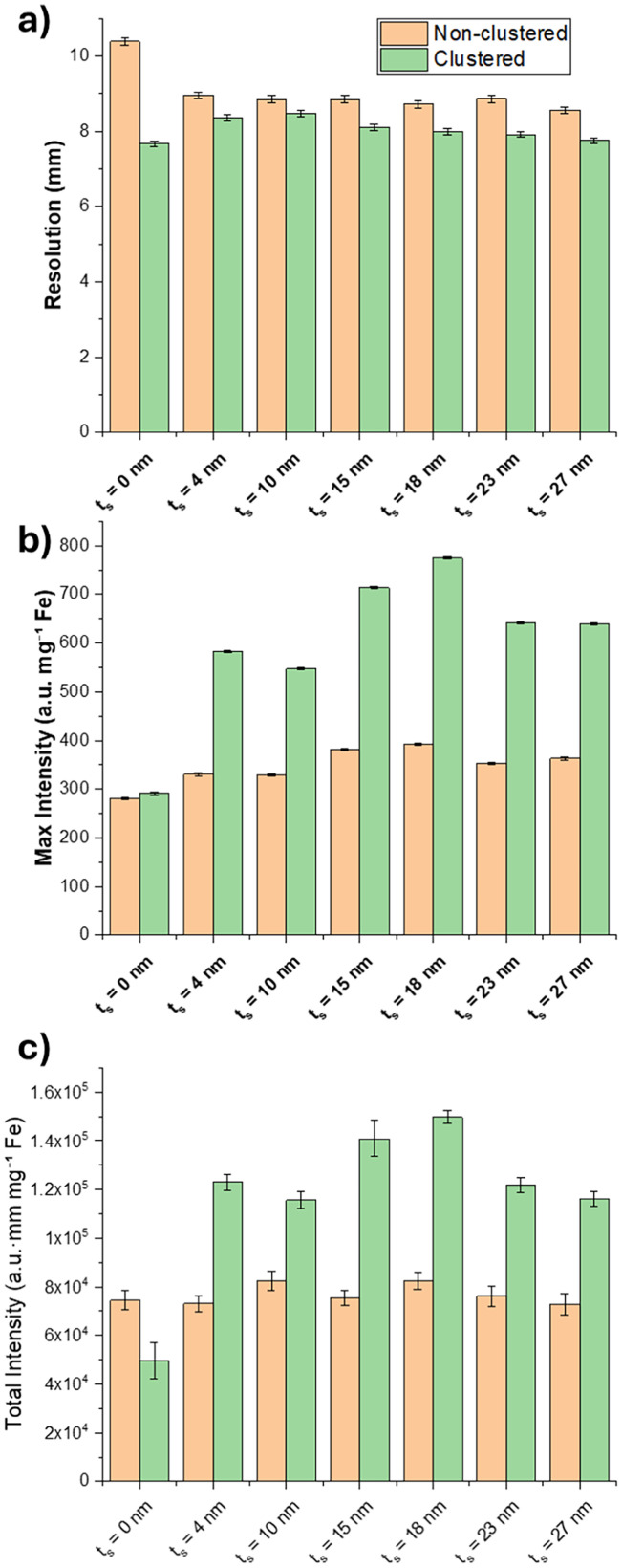
MPI performance metrics non-clustered SPION@SiO_2_ (*t*_S_ = *y* nm) nanoparticles (orange bars) and their clustered analogues SPION@SiO_2_ (*t*_S_ = *y* nm, *C* = 10) (green bars). Charts show MPI data for (a) spatial resolution, (b) maximum signal intensity, and (c) total signal intensity. Error bars represent measurement uncertainty derived from background noise (peak intensity), propagated errors from mean intensity and segmented area (total mean intensity), and Lorentzian fit standard errors with pixel resolution limits (FWHM) (SI Fig. S7 for full images and line profiles).

Fig. 4 shows comparison between the extracted intensity and resolution for non-clustered (orange) and clustered (green) samples. First, we note the improvement in resolution (Fig. 4a) and the significant trend of increasing MPI signal intensity (Fig. 4b and c) and of clustered SPION@SiO_2_ (*t*_S_ = *y* nm, *C* = 10) nanoparticles *vs.* their non-clustered SPION@SiO_2_ (*t*_S_ = *y* nm) analogues, while clustering of the non-silica coated SPION@SiO_2_ (*t*_S_ = 0 nm) showed no improvement in signal intensity. This is consistent with dipolar coupling of silica shell coated SPIONs within the clusters, increasing susceptibility through collective (weakly coupled) reversal, as observed elsewhere.^40,41^ MPI signal generation arises from a combination of Néel (internal magnetic moment reversal, influenced by magnetic anisotropy) and Brownian (whole-particle rotation, influenced by hydrodynamic volume) relaxation processes. As the DLS measurements show comparable *D*_h_ and low PDIs across all clustered samples, variations in Brownian relaxation are unlikely to account for the observed differences in MPI signal intensity. Instead, the dependence of MPI signal enhancement on silica shell thickness is more consistent with changes in interparticle magnetic interactions and Néel relaxation dynamics arising from controlled clustering. This interpretation is supported by the initial Relax™ data discussed earlier, which showed negligible differences in relaxation behaviour as a function of clustering uniformity at fixed silica shell thickness. Taken together, these results indicate that clustering enhances MPI signal through interaction-mediated modification of magnetic relaxation behaviour rather than through changes in overall particle size.

Moving to consider the effect of *t*_S_, we find both resolution and MPI signal generation improve upon introduction of increasing silica layer. This runs counter to the simple model of inter-SPION dipolar coupling, which would naturally decrease as *t*_S_ increases in clustered samples. However, this observation is found in both clustered *and* non-clustered samples, which leads to a likely origin in enhanced passivation of SPION surface spin disorder.^30^

This would have the effect of increasing MPI signal in both non-clustered and clustered samples while also potentially mitigating effects from increasing SPION separation through enhanced stray field coupling from the improved surface spin order.

We attribute this trend primarily to the interplay between surface passivation and changes in dipole–dipole interactions as the inter-core spacing increases. The TEM images in Fig. 3 indicate that for thin silica coatings (*t*_S_ = 4 nm; 10 nm) the shell coverage is less uniform, resulting in regions where the SPION surfaces are only partially passivated. As the silica thickness increases (*t*_S_ = 15 nm; 18 nm), the coating becomes more conformal and uniform. This improved surface coverage is expected to reduce surface spin disorder and enhance magnetic passivation, which is consistent with the observed increase in MPI signal for intermediate shell thicknesses In Fig. 4. However, a decrease in signal is observed for shell thicknesses above ∼18 nm, suggesting that beyond a critical silica thickness the increasing particle size begins to influence the magnetic relaxation behaviour, *e.g.* through increased *D*_h_ and reduced Brownian mobility.

In the clustered systems a similar non-monotonic trend is observed. In addition to the surface passivation effects described above, thicker silica shells reduce the number of magnetic nanoparticles that can be incorporated within clusters of similar *D*_h_, as evident from TEM images (SI Fig. S6). This results in a lower magnetic packing density within the clusters, as well as weaker dipole–dipole coupling as the cores become further separated. Consequently, while intermediate shell thicknesses benefit from improved passivation and cooperative dipolar interactions, further increases in shell thickness dilute the magnetic content of the clusters and reduce the strength of intra-cluster coupling, which likely contributes to the observed reduction in MPI signal. As such, there is an optimal MPI performance at intermediate shell thicknesses, which likely reflects an optimal balance between enhanced surface passivation and dipole–dipole interactions.

To further investigate the role of magnetic particle interactions and silica coating effects, vibrating sample magnetometry (VSM) measurements were performed to directly determine magnetic reversal of the SPIONs. Normalised magnetisation curves (SI Fig. S8) show the variation in hysteresis loop shape upon clustering, with clustered samples exhibiting increased susceptibility and sharper hysteresis loops compared to their non-clustered counterparts. This is indicative of enhanced interparticle dipole–dipole interactions, which promote collective magnetisation reversal behaviour under quasi-static field conditions. Notably, however, the superparamagnetic state is retained with zero remanence magnetisation measured and clusters remaining in suspension throughout measurement.

**Fig. 5:**
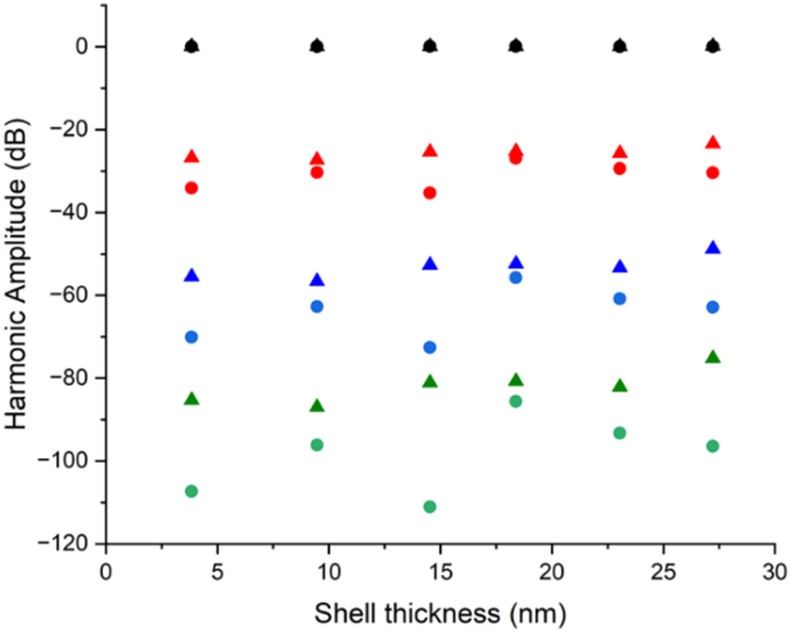
Extracted amplitude *vs.* shell thickness plots for the principal odd harmonics derived from magnetisation curves for non-clustered SPION@SiO_2_ (*t*_S_ = *y* nm) nanoparticles (circles) and their clustered analogues SPION@SiO_2_ (*t*_S_ = *y* nm, *C* = 10) (triangles). Black = fundamental harmonic (normalised to 0 dB), red = 3^rd^ harmonic, blue = 5^th^ harmonic, green = 7^th^ harmonic. For full harmonic signatures see SI Fig. S9.

As an indication of the influence the modified hysteresis loop has on MPI signal, we use the obtained *M*(*H*) curves to model harmonic signal contributions for a 150 Oe drive field MPI experiment, as used in our earlier measurements. Fig. 5 shows the extracted amplitude for the principal odd harmonics in each case (for full harmonic signatures see SI Fig. S9). Higher order harmonics are generated from non-linear saturation and reflect the lower saturation field for the clustered samples, with a pronounced increase in harmonic content compared with the equivalent non-clustered case. This clearly explains the improved MPI signals in Fig. 4 as arising from an increase in cluster susceptibility, due to inter-particle coupling. Revisiting the effect of silica shell thickness, a weak trend is observed of increasing harmonic content in both clustered and non-clustered samples as *t*_S_ increases. This again indicates an improvement in MPI signal with increasing *t*_S_, despite the inter-SPION distance increasing in clustered samples in these cases. Given similar trends are found in both non-clustered and clustered cases, this points to a role of continued passivation from the thickening silica shell. Despite this trend, as shell thickness increases, dipolar interactions in clustered samples remain sufficiently strong to promote cooperative magnetisation switching, while exchange-like coupling is effectively suppressed. In this regime, magnetic moments retain fast, Néel-dominated relaxation dynamics, leading to maximal higher-order harmonic generation under dynamic excitation and, consequently, the brightest MPI signal.

## Conclusions

We have developed a modular synthesis strategy that enables precise control over the spatial organisation of SPIONs within clusters to enhance MPI signal generation. Independent tuning of silica shell thickness allows for the systematic control of inter-core separation during cluster assembly, promoting cooperative magnetisation switching through dipole–dipole coupling while suppressing potential exchange-like interactions. This surfactant-mediated assembly and modular silica-spacer strategy provides a facile route to tune interparticle separation, offering a versatile platform for next-generation MPI tracer development. The interplay between the different potential conflicting effects, *i.e.*, interparticle magnetic interactions, surface spin passivation, and magnetic relaxation dynamics, establishes clustered SPION@SiO_2_ systems as a complex yet inviting platform for precisely tuning and optimising MPI performance. While the trends observed here are consistent with changes in surface passivation and interparticle magnetic coupling, further magnetic characterisation (*e.g.*, remanence magnetisation analysis or blocking temperature measurements) would be valuable to quantitatively resolve the relative contributions of these mechanisms, thus warranting continued investigation.

## Author contributions

EUH, L'OB and MG designed the experiments and wrote the manuscript. EUH conducted all synthetic work. EUH, AM and LO conducted magnetic and material characterization. EUH and MM conducted all MPI imaging acquisition and imaging analysis. All authors contributed to data interpretation and the writing of the article.

## Conflicts of interest

There are no conflicts to declare.

## Supplementary Material

NR-018-D6NR00223D-s001

## Data Availability

The data supporting the findings of this study are available within the article and/or its supplementary information (SI). Supplementary information: TEM images with histograms, XRD plots, Relax data, DLS data, 2D MPI images, VSM data. See DOI: https://doi.org/10.1039/d6nr00223d. The MATLAB code created by EUH supporting this study is available at https://doi.org/10.5281/zenodo.15777147.

## References

[cit1] Lapusan R., Borlan R., Focsan M. (2024). Nanoscale Adv..

[cit2] Palanisamy S., Wang Y. M. (2019). Dalton Trans..

[cit3] Pucci C., Degl’Innocenti A., Gümüş M. B., Ciofani G. (2022). Biomater. Sci..

[cit4] Gleich B., Weizenecker J. (2005). Nature.

[cit5] Bauer L. M., Situ S. F., Griswold M. A., Samia A. C. S. (2015). J. Phys. Chem. Lett..

[cit6] Weizenecker J., Gleich B., Rahmer J., Dahnke H., Borgert J. (2009). Phys. Med. Biol..

[cit7] Harvell-Smith S., Tunga L. D., Thanh N. T. K. (2021). J. Phys. Chem. Lett..

[cit8] Duong H. T. K., Abdibastami A., Gloag L., Barrera L., Gooding J. J., Tilley R. D. (2022). Nanoscale.

[cit9] Bishop K. J. M., Wilmer C. E., Soh S., Grzybowski B. A. (2009). Small.

[cit10] Dušak P., Mertelj A., Kralj S., Makovec D. (2014). J. Colloid Interface Sci..

[cit11] Majetich S. A., Wen T., Booth R. A. (2011). ACS Nano.

[cit12] Grzelczak M., Vermant J., Furst E. M., Liz-Marzán L. M. (2010). ACS Nano.

[cit13] Kavre I., Kostevc G., Kralj S., Vilfan A., Babič D. (2014). RSC Adv..

[cit14] Hickey R. J., Haynes A. S., Kikkawa J. M., Park S. J. (2011). J. Am. Chem. Soc..

[cit15] Di Corato R., Bigall N. C., Ragusa A., Dorfs D., Genovese A., Marotta R., Manna L., Pellegrino T. (2011). ACS Nano.

[cit16] Pöselt E., Kloust H., Tromsdorf U., Janschel M., Hahn C., Maßlo C., Weller H. (2012). ACS Nano.

[cit17] Avugadda S. K., Wickramasinghe S., Niculaes D., Ju M., Lak A., Silvestri N., Nitti S., Roy I., Samia A. C. S., Pellegrino T. (2021). Nanomaterials.

[cit18] Moor L., Scheibler S., Gerken L., Scheffler K., Thieben F., Knopp T., Herrmann I. K., Starsich F. H. L. (2022). Nanoscale.

[cit19] Ureña Horno E., Maguire M. L., Ozkan S., O'Brien L., Murray P., Poptani H., Giardiello M. (2025). Nanoscale.

[cit20] Hansen M. F., Mørup S. (1998). J. Magn. Magn. Mater..

[cit21] Rosensweig R. E. , Ferrohydrodynamics, Dover Publications, Inc., New York, 2014

[cit22] Mørup S., Hansen M. F., Frandsen C. (2010). Beilstein J. Nanotechnol..

[cit23] Wu K., Su D., Saha R., Liu J., Wang J. P. (2019). J. Phys. D: Appl. Phys..

[cit24] García-Acevedo P., Piñeiro Y., Gallo J., Ramos-Cabrer P., Iglesias-Rey R., Rivas J., Bañobre-López M. (2026). Adv. Sci..

[cit25] Zhou Z., Tian R., Wang Z., Yang Z., Liu Y., Liu G., Wang R., Gao J., Song J., Nie L., Chen X. (2017). Nat. Commun..

[cit26] Vogt C., Toprak M. S., Muhammed M., Laurent S., Bridot J.-L., Müller R. N. (2010). J. Nanopart. Res..

[cit27] Zhang M., Cushing B. L., O'Connor C. J. (2008). Nanotechnology.

[cit28] Pereira M., Pereira C., Silva A. S., Schmool D. S., Freire C., Grenche J. M., Arajo J. P. (2011). J. Appl. Phys..

[cit29] Coşkun M., Korkmaz M. (2014). J. Nanopart. Res..

[cit30] De Toro J. A., Vasilakaki M., Lee S. S., Andersson M. S., Normile P. S., Yaacoub N., Murray P., Sánchez E. H., Muñiz P., Peddis D., Mathieu R., Liu K., Geshev J., Trohidou K. N., Nogués J. (2017). Chem. Mater..

[cit31] de Mendonça E. S. D. T., de Faria A. C. B., Dias S. C. L., Aragón F. F. H., Mantilla J. C., Coaquira J. A. H., Dias J. A. (2019). Surf. Interfaces.

[cit32] Khandhar P., Ferguson R. M., Arami H., Krishnan K. M. (2013). Biomaterials.

[cit33] Goodwill P. W., Conolly S. M. (2010). IEEE Trans. Med. Imaging.

[cit34] Nigam S., Mohapatra J., Makela A. V., Hayat H., Rodriguez J. M., Sun A., Kenyon E., Redman N. A., Spence D., Jabin G., Gu B., Ashry M., Sempere L. F., Mitra A., Li J., Chen J., Wei G. W., Bolin S., Etchebarne B., Liu J. P., Contag C. H., Wang P. (2024). Small.

[cit35] Park J., An K., Hwang Y., Park J.-G., Noh H.-J., Kim J.-Y., Park J.-H., Hwang N.-M., Hyeon T. (2004). Nat. Mater..

[cit36] Giardiello M., Hatton F. L., Slater R. A., Chambon P., North J., Peacock A. K., He T., Mcdonald T. O., Owen A., Rannard S. P. (2016). Nanoscale.

[cit37] Ureña-Horno E., Kyriazi M. E., Kanaras A. G. (2021). Nanoscale Adv..

[cit38] Ding H. L., Zhang Y. X., Wang S., Xu J. M., Xu S. C., Li G. H. (2012). Chem. Mater..

[cit39] Bai F., Wang D., Huo Z., Chen W., Liu L., Liang X., Chen C., Wang X., Peng Q., Li Y. (2007). Angew. Chem., Int. Ed..

[cit40] Them K. (2017). Phys. Med. Biol..

[cit41] Bender P., Wetterskog E., Honecker D., Fock J., Frandsen C., Moerland C., Bogart L. K., Posth O., Szczerba W., Gavilán H., Costo R., Fernández-Díaz M. T., González-Alonso D., Fernández Barquín L., Johansson C. (2018). Phys. Rev. B.

