# Supplementary material for: Tailoring inter-core distance of clustered SPIONs using silica spacers for enhanced magnetic particle imaging (MPI)
Source: Nanoscale. 2026 Apr 9;18(19):10029–35. doi: 10.1039/d6nr00223d (PMC13089672; doi:10.1039/d6nr00223d)
Supplement: NR-018-D6NR00223D-s001 [file NR-018-D6NR00223D-s001.pdf]

Supplementary Information (SI) for Nanoscale.
This journal is © The Royal Society of Chemistry 2026

Tailoring Inter-Core Distance of Clustered SPIONs Using Silica Spacers for Enhanced Magnetic Particle Imaging (MPI)

Elena Ureña Horno,[a*] Alexi Mores,[b] Lewis Owens,[a] Mahon L. Maguire,[c] Harish Poptani,[c,d] Liam O'Brien[b] and Marco Giardiello[a**]

Supplementary Information

		Pages
	Materials and Methods	2-3
	Characterization	4-5
**Figure S1**	TEM images (with histograms) and XRD data for 15 ± 3 nm SPIONs and SPION@SiO$_2$(t_S=20 nm) nanoparticles	6
**Figure S2**	DLS traces for non-clustered SPION@SiO$_2$(t_S=20 nm) and clustered SPION@SiO$_2$(t_S=20 nm, C=x) nanoparticles	7
**Figure S3**	Relax™ relaxation data showing overlaid point spread functions (PSF) for non-clustered SPION@SiO$_2$(t_S=20 nm) and clustered SPION@SiO$_2$(t_S=20 nm, C=x) nanoparticles	7
**Figure S4**	TEM images (with histograms) and XRD data for 17.24 ± 1.43 nm SPIONs	8
**Figure S5**	DLS traces for clustered SPION@SiO$_2$ (t_S=y nm, C=10) nanoparticles	9
**Figure S6**	TEM images of clustered SPION@SiO$_2$ (t_S=y nm, C=10)	10
**Figure S7**	2D MPI images of non-clustered SPION@SiO$_2$ (t_S=y nm) nanoparticles and their clustered analogues SPION@SiO$_2$ (t_S=y nm, C=10)	11-12
**Figure S8**	VSM curves for of non-clustered SPION@SiO$_2$ (t_S=y nm) nanoparticles and their clustered analogues SPION@SiO$_2$ (t_S=y nm, C=10).	13
**Figure S9**	Harmonic profiles taken VSM curves for non-clustered SPION@SiO$_2$ (t_S=y nm) nanoparticles and their clustered analogues SPION@SiO$_2$ (t_S=y nm, C=10).	14

Materials and Methods

Materials

Oleic acid (OA), 1-octadecene (ODE), iron chloride hexahydrate ($FeCl_3·6H2O$), sodium oleate, polyoxyethylene (5) nonylphenylether (IGEPAL CO-520, average Mn 441), tetraethyl orthosilicate (TEOS, ≥ 99%), and cetyltrimethyl ammonium bromide (CTAB, ≥ 99%) were purchased from Sigma-Aldrich. Ammonia Solution (35%) was purchased from Thermo-Fisher. All chemicals were used as received without further purification.

Synthesis of Superparamagnetic Iron Oxide Nanoparticles (SPION)

SPIONs were synthesised through thermal decomposition, first by preparing an iron oleate precursor. Preparation of Iron oleate precursor: In a 100 mL three-neck round-bottomed flask, $FeCl_3·6H_2O$ (8 mmol, 2.162 g) and sodium oleate (24 mmol, 7.308 g) were mixed with 24 mL of H_2O and 16 mL of ethanol. The solution was then heated to reflux under the presence of N_2 gas for 4 h. After that, the solution was cooled to room temperature. The iron oleate precursor was then extracted with H_2O and hexane and dried in a drying oven. Synthesis of SPIONs: In a 50 mL three-neck round bottom flask, 900 mg (1 mmol) of iron oleate precursor was mixed with 159 µL of OA and 15 mL of ODE under N_2 gas flow. The solution was heated to reflux (320°C) and maintained at this temperature for 30 min. The solution was then allowed to cool down naturally to room temperature, and SPIONs were precipitated by centrifugation and washed with ethanol three times (10000 rpm, 30 min). SPIONs were then re-dispersed in hexane at 10 mg/mL and stored for further use.

Silica Coating of SPIONs; SPION@SiO_2

Silica shells were deposited on the hydrophobic SPION cores using a reverse microemulsion technique. 0.5 g of Igepal CO-520 was dissolved in hexane A reverse (water-in-oil) microemulsion was then formed by adding ammonium hydroxide solution (35%) under vigorous stirring, followed by addition of TEOS; varied volumes of both were added to control silica shell thickness (see Table S1). Then, 10 mg of hydrophobic SPION cores dispersed in hexane (10 mg/mL) were introduced into the microemulsion and the reaction was left at room temperature for 24 hours. SPION@SiO_2 nanoparticles were isolated by centrifugation, washed with ethanol and water, and redispersed in ethanol at 10 mg/mL for further processing.

Table S1. Summary of synthesis parameters for SPION@SiO_2 nanoparticles prepared *via* reverse microemulsion. t_S= shell thickness (nm). Errors reported as ± standard deviation across 100 particles measure by TEM.

Sample	SPION (mg)	Igepal CO-520 (g)	NH_4OH (µL)	TEOS (µL)	Silica Thickness (nm)	Final Diameter (nm ± SD)
SPION@SiO_2(t_S=20 nm)	10	0.5	100	100	20.0 ± 3	55 ± 6
SPION@SiO_2(t_S=4 nm)	10	0.5	50	25	3.84 ± 1.2	24.92 ± 1.83
SPION@SiO_2(t_S=10 nm)	10	0.5	100	50	9.47 ± 1.5	36.18 ± 2.56
SPION@SiO_2(t_S=15 nm)	10	0.5	100	75	14.53 ± 1.3	46.30 ± 2.09
SPION@SiO_2(t_S=18 nm)	10	0.5	100	100	18.39 ± 1.2	54.01 ± 2.28
SPION@SiO_2(t_S=23 nm)	10	0.5	100	150	23.05 ± 1,2	64.50 ± 1.83
SPION@SiO_2(t_S=27 nm)	10	0.5	100	300*	27.22 ± 1.2	71.67 ± 1.96

*Sample SPION@SiO_2(t_S=27 nm) involved TEOS addition in two sequential portions (150 µL + 150 µL)

Clustering of Silica-coated SPIONs

Clusters of silica-coated SPIONs were synthesized via a surfactant-mediated assembly. 1 mL of silica-coated nanoparticles suspended in ethanol (10 mg/mL) were mixed with 10 mL of aqueous solutions of CTAB at varying concentrations (Table S2). The mixture was sonicated using a sonication bath for 4 hours under gentle heating to facilitate ethanol evaporation and promote self-assembly of SPION cores into clusters within CTAB micelles.

Table 2. CTAB masses and concentrations employed for SPION@SiO_2(t_S=20 nm, C=x) clustering optimisation

Sample	CTAB (mg)	CTAB (mM)
SPION@SiO_2(t_S=20 nm, C=2.5)	2.5	0.686
SPION@SiO_2(t_S=20 nm, C=5)	5	1.37
*SPION@SiO_2(t_S=20 nm, C=10)	10	2.74
SPION@SiO_2(t_S=20 nm, C=20)	20	5.49

SPION@SiO_2(t_S=20 nm, C=30)	30	8.23

*CTAB concentation used for all SPION@SiO_2(t_S=y nm, C=10) samples

Characterization

Transmission Electron Microscopy (TEM)

Samples for TEM imaging were prepared by drop-casting dilute dispersions of nanoparticles or clusters onto carbon-coated copper grids, followed by solvent evaporation under ambient conditions. Images were acquired using a FEI Tecnai with an operating voltage of 120 kV. Core sizes, silica shell thicknesses, and cluster morphologies were quantified using ImageJ software measuring over 100 particles for the statistical analysis, with size distributions reported as mean values ± standard deviation.

Dynamic Light Scattering (DLS)

Hydrodynamic size distributions, D_h, of nanoparticle in suspension were measured with a Malvern Zetasizer Nano ZS equipped with a 4 mW He-Ne 633 nm laser. Samples were diluted to appropriate concentrations to avoid multiple scattering effects and equilibrated at 25 °C prior to measurement. Scans were run three times, with errors quoted as ± standard deviation.

X-ray diffraction (XRD)

XRD measurements were performed using a Bruker diffractometer equipped with a Co Kα radiation source (λ = 1.78901 Å) operated in transmission mode using a stainless-steel sample holder. Data were collected over a scan duration of 2 hours. XRD patterns were assigned using the Crystallography Open Database for magnetite Fe_3O_4 (COD, entry 9006189).

Inductively Coupled Plasma Optical Emission Spectroscopy (ICP-OES).

Fe(III) concentrations were determined by ICP-OES using an Agilent Technologies 5110 ICP-OES instrument. Prior to analysis, samples containing silica-coated materials were digested using a HF/HNO_3 mixture to ensure complete dissolution of the silica shells. Aliquots of sample were transferred to acid-resistant digestion vessels and treated with concentrated hydrofluoric acid (HF, 48% w/w) and concentrated nitric acid (HNO_3, 69–70% w/w) in a 2:1 (v/v) $HF:HNO_3$ ratio. The mixtures were allowed to react until digestion was complete, after which the solutions were diluted into nitric acid to reduce residual HF to negligible levels, and the digests were diluted to volume with 2% (v/v) HNO_3 prior to ICP-OES measurement.

Relax™ and Magnetic Particle Imaging (MPI) measurements

MPI experiments were performed using the Momentum™ MPI system (Magnetic Insight Inc.). For 2D imaging and Relax™ relaxometry data analysis, samples of known iron concentration and fixed volumes (400 mL) were loaded into custom holders[19] and imaged under identical

acquisition parameters, where the system was operated at a fixed frequency of 45 kHz, with drive field amplitudes of 20 and 26 mT along the x and z axes, respectively. The 120 mm (Z)× 60 mm (X) field of view (FOV) was centred on the sample. For Relax™ relaxometry data analysis, the FWHM of the PSF was measured in Tesla (T). The MPI resolution (mm) was estimated by dividing the FWHM of the PSF by the imaging gradient strength, 5.7 T/m, operating in the standard mode. For MPI 2D imaging, the magnetic field gradient was set to 3 T/m. To evaluate quantitative sensitivity and resolution, the data is reported as both the Maximum Intensity value and the Total Intensity value; maximum intensity was defined as the peak signal value detected within the image, while the total intensity was determined by multiplying the mean signal intensity within the defined region of interest (ROI) by the corresponding ROI area (in mm^2). Spatial resolution was determined by calculating the full width at half maximum (FWHM) along the x-axis. For this, a line profile was extracted through the voxel exhibiting the highest intensity, fitted to a Lorentzian distribution, from which the FWHM (in millimetres) was derived. ROI extraction and signal quantification were performed using our custom, user-friendly MATLAB script to accelerate MPI image analysis. The script is freely accessible online at https://doi.org/10.5281/zenodo.15777147.

Vibrating Sample Magnetometry (VSM)

Magnetic measurements were performed using a Cryogenic Ltd vibrating sample magnetometer (VSM). Aliquots of dispersed sample were loaded into a sealed non-magnetic liquid sample holder (supplied by Cryogenic Ltd), ensuring no headspace or leakage during vibration. Scans were recorded at 300K in a field regime of ± 2 T using a ramp rate of 0.1 T/min, but with a ramp rate of 0.01 T/min up to ± 0.5 T for improved resolution on the shape of the hysteresis curve.

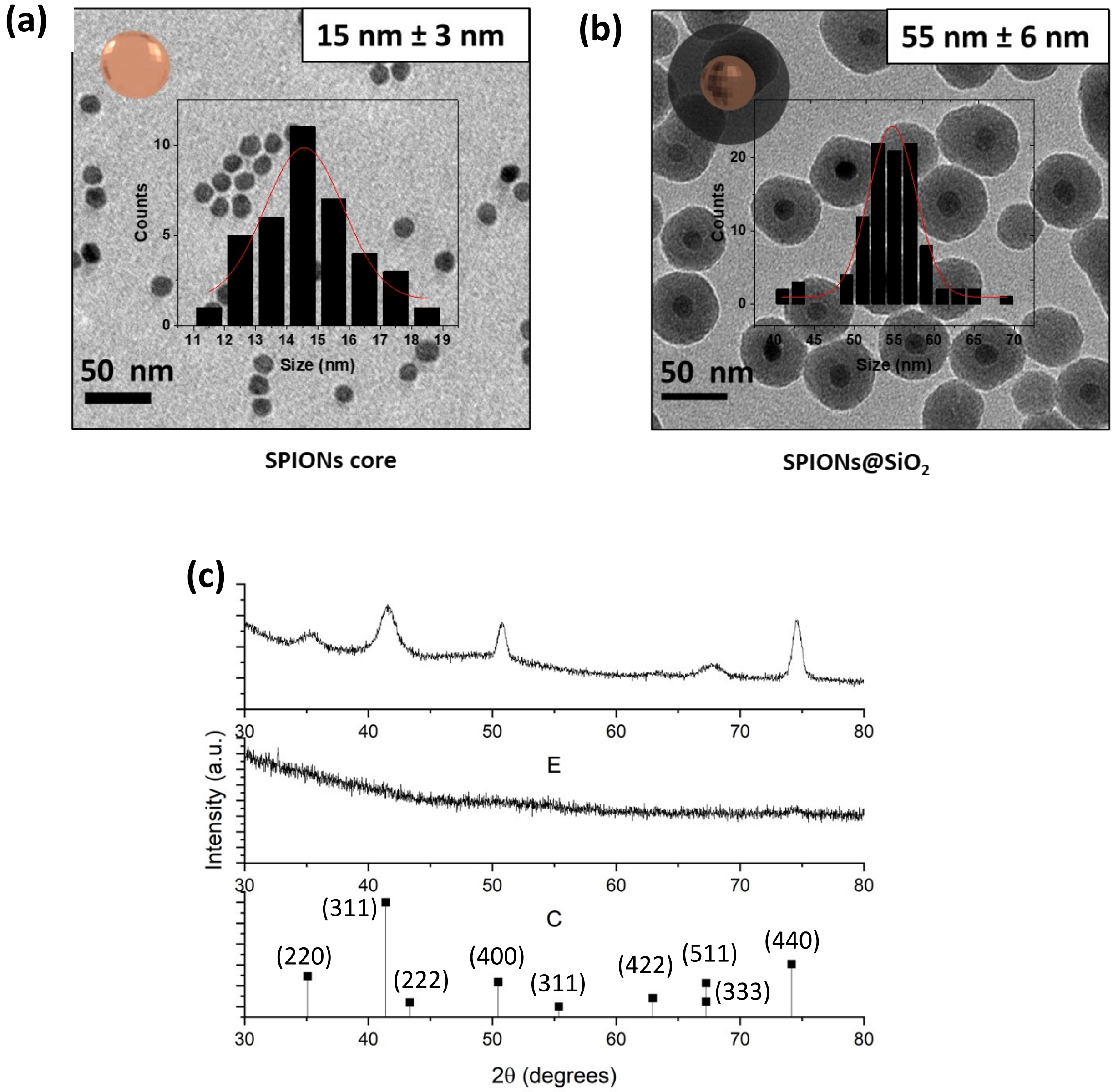

Figure S1. (a) Representative TEM image of as-synthesized SPION cores with an average diameter of approximately 15 ± 3 nm. (b) TEM image of SPION@SiO$_2$(t_S=20 nm), coated with a uniform silica shell (~20 ± 2 nm thickness), resulting in core–shell nanoparticles with an overall diameter of 55 ± 6 nm. Particle size distribution histograms (inset) was generated using ImageJ software measuring over 100 particles for the statistical analysis, with errors reported as ± standard deviation. (c) XRD plots of (i) SPIONs and (ii) (SPION@SiO$_2$(t_S=20 nm), (iii) reference taken from the Crystallography Open Database (COD - 9006189) for magnetite Fe3O$_4$ and adapted to the Co Kα1 radiation (λ = 1.789010 Å) used in our XRD equipment.

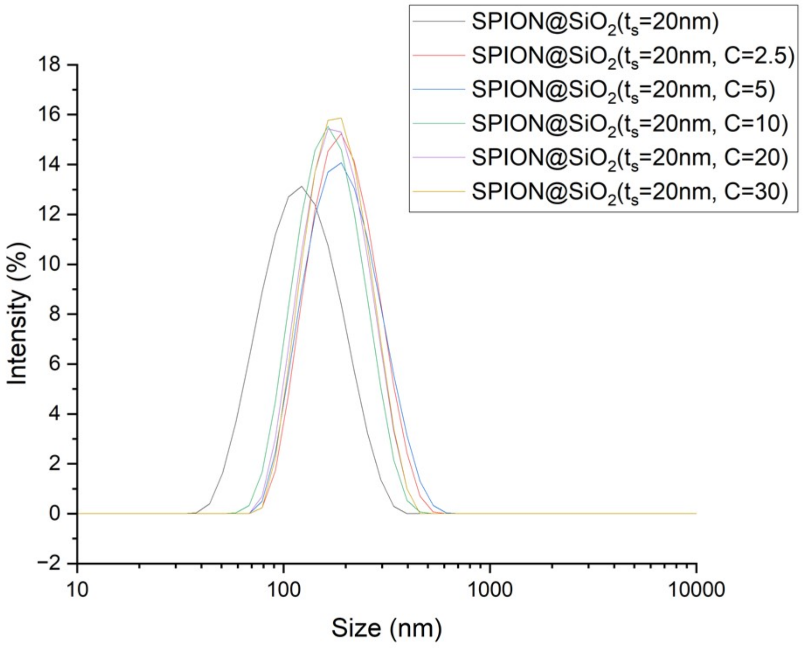

Figure S2. DLS traces for non-clustered SPION@SiO_2(t_S=20 nm) and clustered SPION@SiO_2(t_S=20 nm, C=x) nanoparticles, where c= CTAB concentration (mM) employed.

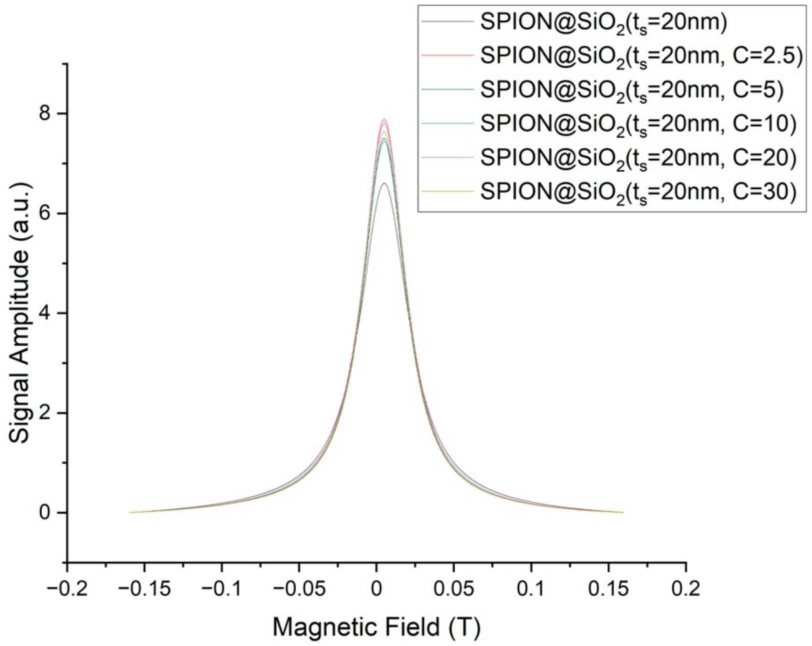

Figure S3. Relax™ relaxation data showing overlaid point spread functions (PSF) for non-clustered SPION@SiO_2(t_S=20 nm) and clustered SPION@SiO_2(t_S=20 nm, C=x) nanoparticles. Peak height is quited for signal intentsity, resolution (mm) was estimated by dividing the FWHM of the PSF by the imaging gradient strength, 5.7 T/m

(a)

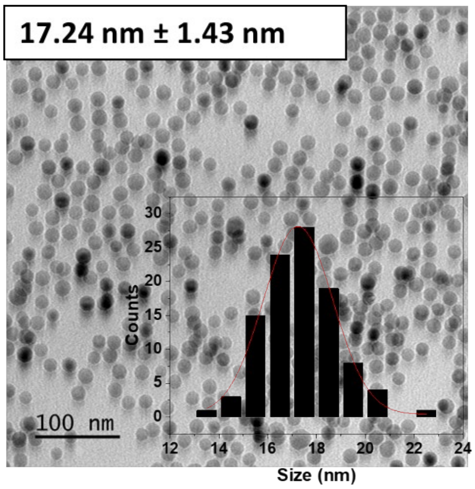

(b)

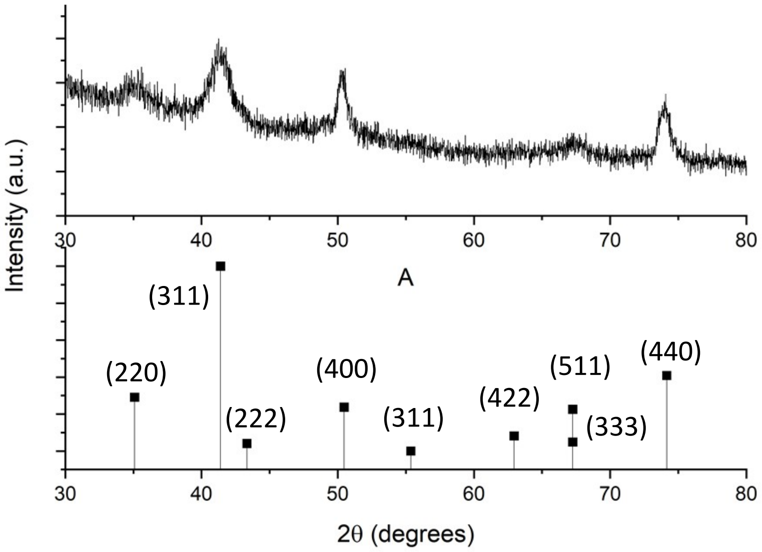

Figure S4. (a) Representative TEM image of synthesized SPION cores. Particle size distribution histograms (inset) was generated using ImageJ software measuring over 100 particles for the statistical analysis with errors reported as standard deviation, showing size distribution of SPION cores with an average diameter of 17.24 ± 1.43 nm. (b) XRD plots of (i) SPIONs, (ii) reference taken from the Crystallography Open Database (COD - 9006189) for magnetite Fe3O_4 and adapted to the Co Kα1 radiation (λ = 1.789010 Å) used in our XRD equipment.

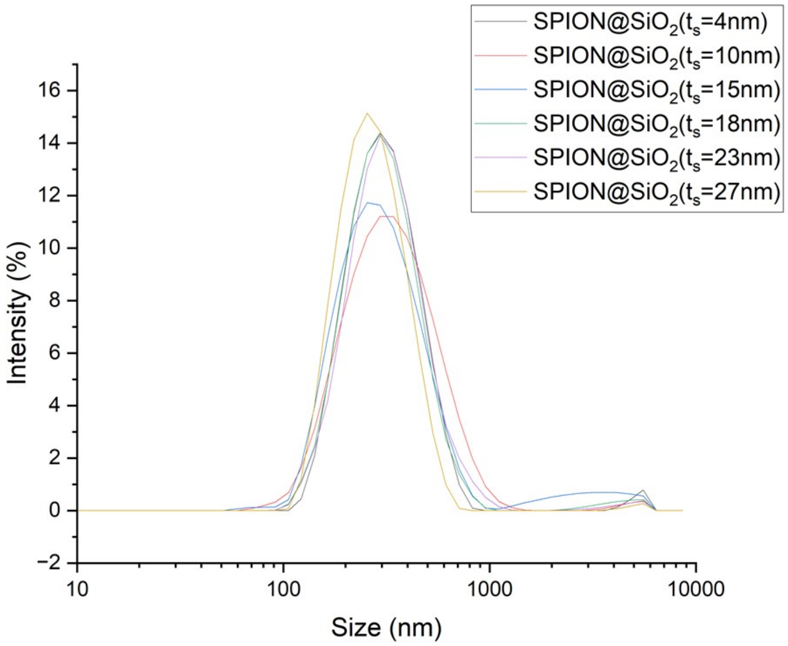

Figure S5. DLS traces for clustered *SPION@SiO₂ (t_S=y nm, C=10)* nanoparticles, where t_s = silica shell thickness.

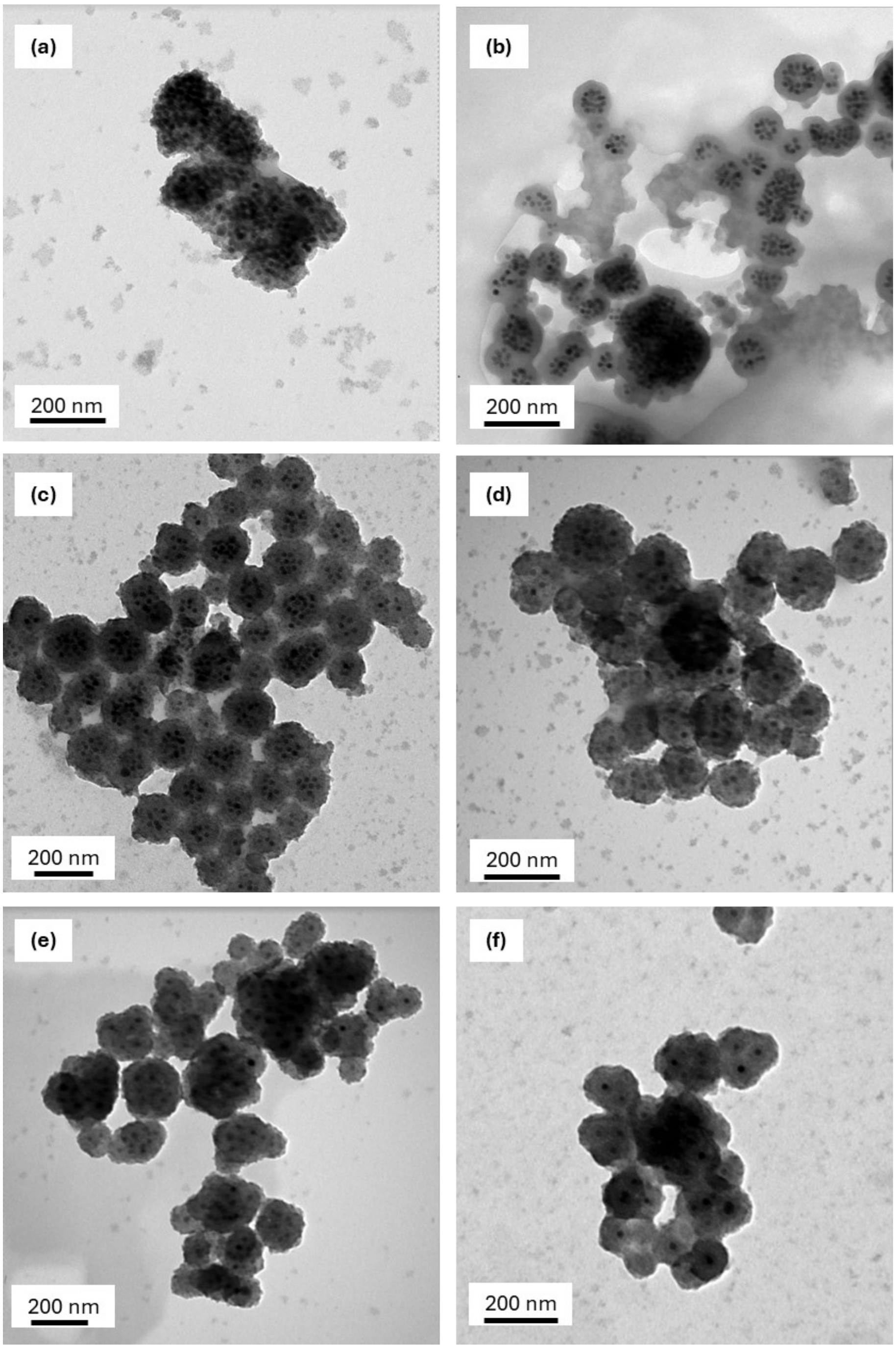

Figure S6. Representative TEM images of clustered SPION@SiO₂ (t_S=y nm, C=10), where t_S = **(a)** 4 nm, **(b)** 10 nm, **(c)** 15 nm, **(d)** 18 nm, **(e)** 23 nm, and **(f)** 27 nm.

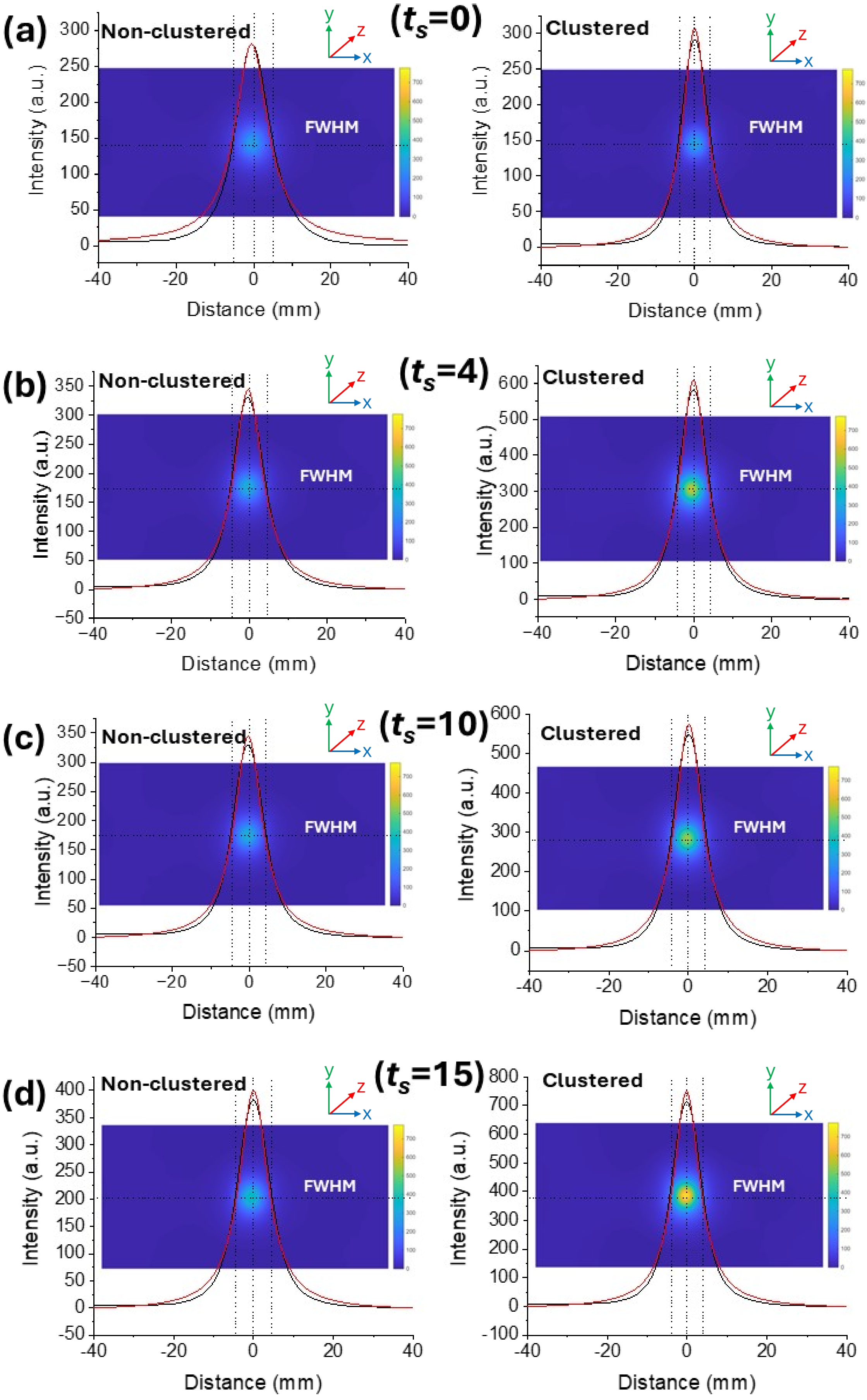

(Figure and caption continues next page)

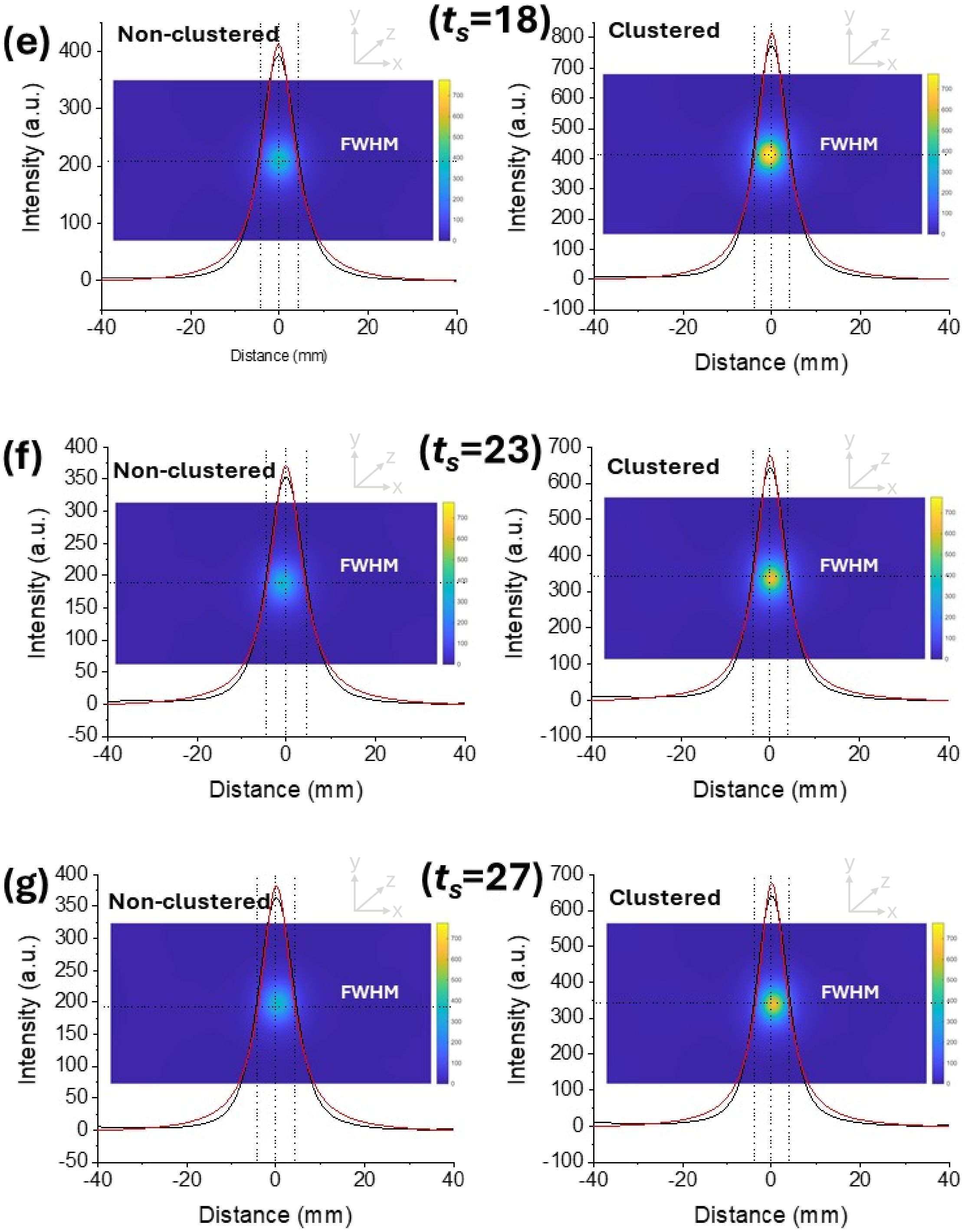

Figure S7. 2D MPI images of non-clustered SPION@SiO_2 (t_S=y nm) nanoparticles and their clustered analogues SPION@SiO_2 (t_S=y nm, C=10). Images are scaled to same intensity (700 nm) for consistent visual comparison. Line profiles extracted horizontally across each image (black lines) are overlaid with the corresponding Lorentzian fits (red lines). Line profiles scled to maximum signal value. Image pairs correspond to silica shell thicknesses, t_S = **(a)** 0 nm, **(b)** 4 nm, **(c)** 10 nm, **(d)** 15 nm, **(e)** 18 nm, **(f)** 23 nm and **(g)** = 27 nm.

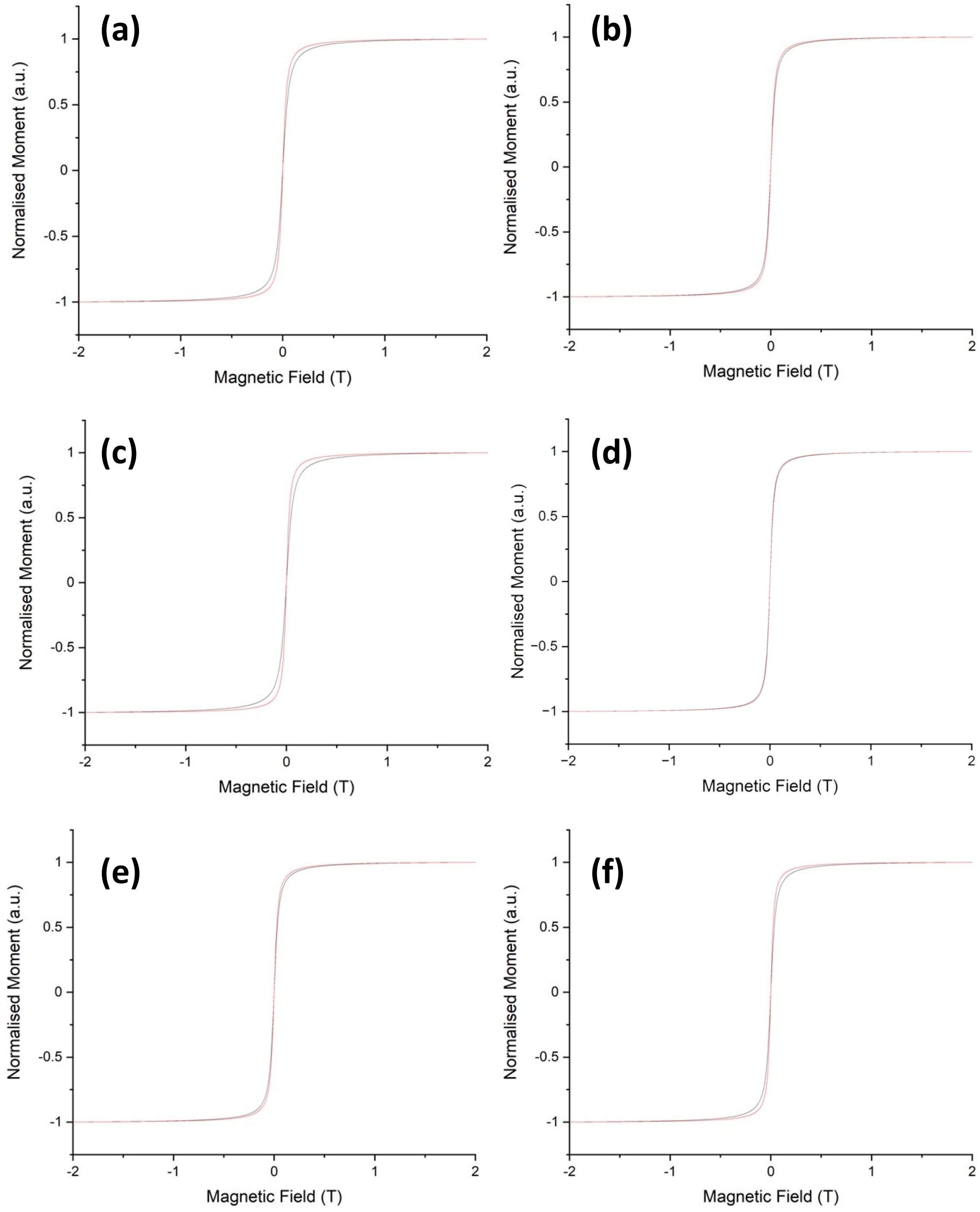

Figure S8. VSM curves for non-clustered SPION@SiO_2 (t_S=y nm) nanoparticles (black lines) and their clustered analogues SPION@SiO_2 (t_S=y nm, C=10) (red lines). Charts correspond to silica shell thicknesses, t_S = **(a)** 4 nm, **(b)** 10 nm, **(c)** 15 nm, **(d)** 18 nm, **(e)** 23 nm and **(f)** = 27 nm.

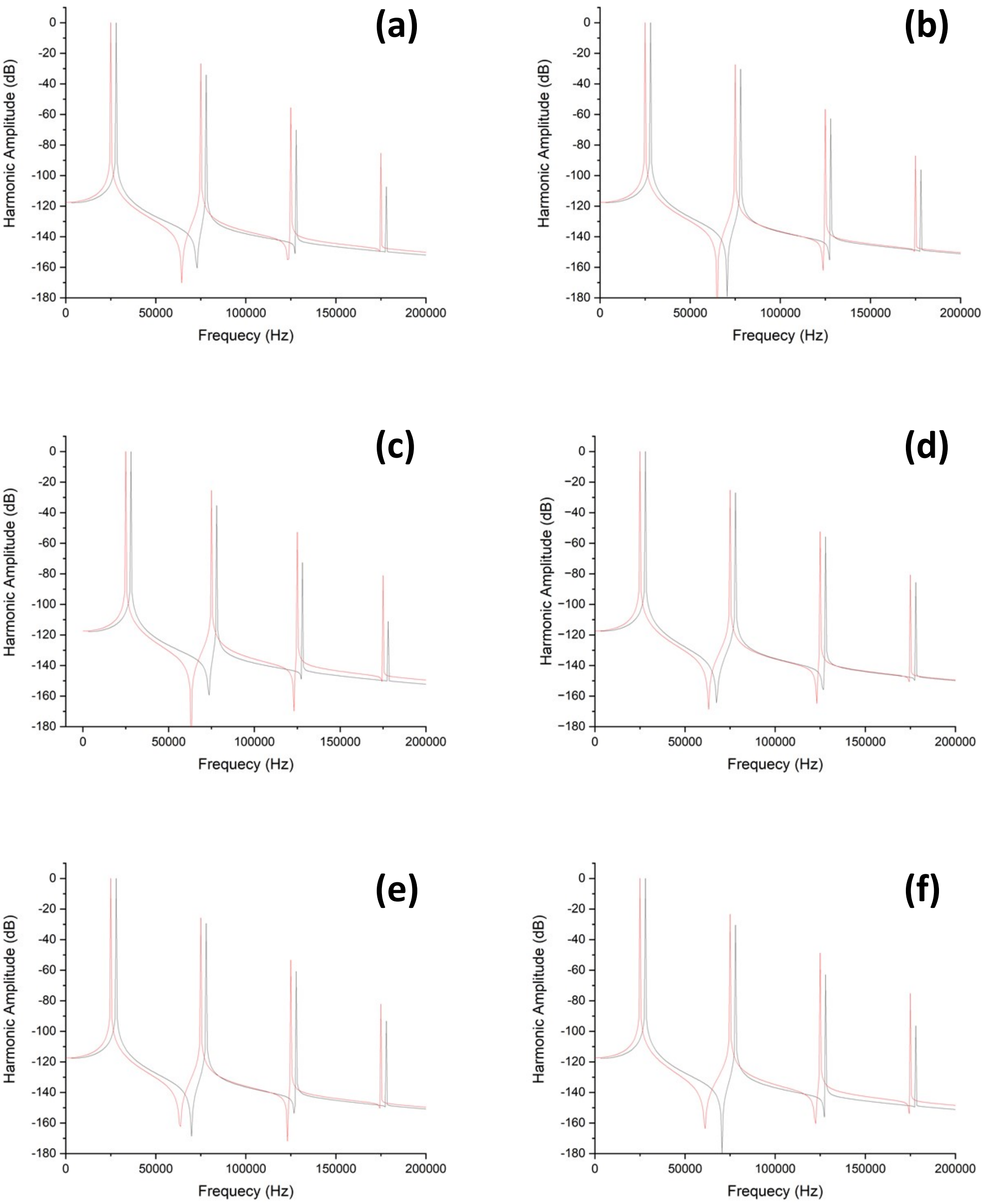

Figure S9. Harmonic profiles taken VSM curves for of non-clustered SPION@SiO_2 (t_S=y nm) nanoparticles (black lines) and their clustered analogues SPION@SiO_2 (t_S=y nm, C=10) (red lines). Charts correspond to silica shell thicknesses, t_S = **(a)** 4 nm, **(b)** 10 nm, **(c)** 15 nm, **(d)** 18 nm, **(e)** 23 nm and **(f)** = 27 nm. (Note, black lines offset by 3000Hz to enable side by side comparison).
